# Reliability and Validity of Ultrasound in Identifying Anatomical Landmarks for Diagnosing A2 Pulley Ruptures: A Cadaveric Study

**DOI:** 10.3390/diagnostics14192149

**Published:** 2024-09-27

**Authors:** Xeber Iruretagoiena, Volker Schöffl, Ramón Balius, Marc Blasi, Fernando Dávila, Xavier Sala-Blanch, Asier Dorronsoro, Javier de la Fuente

**Affiliations:** 1 Deusto Physical TherapIker, Physical Therapy Department, Faculty of Health Sciences, University of Deusto, 20012 San Sebastiän, Spain; 2 Eskura Osasun Zentroa, 20200 Beasain, Spain; asierdorronsoro@outlook.com; 3 Sputnik Investigación, 28232 Madrid, Spain; 4 Section Sportsorthopedics and Sportsmedicine, Department of Orthopedic and Trauma Surgery, Klinikum Bamberg, 96049 Bamberg, Germany; volker.schoeffl@me.com; 5 Department of Trauma Surgery, Friedrich Alexander University of Erlangen-Nuremberg, 91054 Erlangen, Germany; 6 Section of Wilderness Medicine, Department of Emergency Medicine, University of Colorado School of Medicine, Denver, CO 80045, USA; 7 School of Clinical and Applied Sciences, Leeds Becket University, Leeds LS1 3HE, UK; 8 Consell Catala de l’Esport, Generalitat de Catalunya, 08950 Barcelona, Spain; ramonbaliusmatas@gmail.com; 9 Sport Medicine and Imaging Department, Clínica Diagonal, 08950 Barcelona, Spain; 10 Department of Plastic Surgery, Hospital Germans Trias I Pujol, 08916 Barcelona, Spain; marcblasibrugue@gmail.com; 11 Orthopedics Department, Hospital Bidasoa, 20280 Irun, Spain; fdavila.cot@gmail.com; 12 Anatomy and Embryology Department, School of Medicine, Universitat de Barcelona, 08007 Barcelona, Spain; xavi.sala.blanch@gmail.com; 13 Department of Anesthesiology, Hospital Clínic de Barcelona, 08036 Barcelona, Spain; 14 Orthopedics Department, Clínica Pakea-Mutualía, 20018 San Sebastiän, Spain; jfuenteortiz@gmail.com

**Keywords:** pulley, ultrasonography, anatomical landmarks, tendon–bone distance, phalanx

## Abstract

**Background/Objectives**: Rock climbing is becoming more popular, leading to an increased focus on diagnosing and treating related injuries. Finger pulley and flexor tendon injuries are common among climbers, with the A2 pulley being the most frequently affected. High-resolution ultrasound (US) is the preferred method for detecting pulley injuries. This study aimed to determine the reliability and validity of US in identifying anatomical landmarks for diagnosing A2 pulley ruptures. **Methods**: This study was cross-sectional, involving 36 fingers from 4 cadaver arms. A Canon Aplio i800 US machine was used to measure two anatomical landmarks: the midpoint of the proximal phalanx and the distal edge of the A2 pulley. For the first anatomical landmark, the length of the proximal phalanx (PP distance), and for the second landmark, the distance between the distal edges of the proximal phalanx and the A2 pulley (“A” distance), were measured. Measurements were performed by two sonographers and compared to a digital caliper measurement taken post-cadaver dissection. Observers were blinded during measurements to ensure unbiased results. **Results**: Overall PP distance measured by US (O1: 37.5 ± 5.3 mm, O2: 37.8 ± 5.4 mm) tended to be shorter than caliper measurements (O3: 39.5 ± 5.5 mm). The differences between sonographers were minimal, but larger when compared to caliper measurements. High reliability for PP distance measurement was observed, especially between sonographers, with an ICC average of 0.99 (0.98, 1.00). However, reliability was lower for the “A” distance, with significant differences between US and caliper measurements. Regarding validity, US measurements were valid when compared to caliper measurements for PP distance, but not as reliable for the “A” due to wider confidence intervals. While US can substitute caliper measurements for PP distance (LR, Y:O2, X:O3, −0.70 (−3.28–1.38), 0.98 (0.93 ± 1.04)), its validity for “A” distance is lower (LR, Y:O2, X:O3, −2.37 (−13.53–4.83), 1.02 (0.62–1.75)). **Conclusions**: US is a reliable and valid tool in identifying anatomical landmarks for diagnosing A2 pulley ruptures, particularly for detecting the midpoint of the proximal phalanx. This is important to differentiate between complete and partial A2 pulley tears. However, the measurement of the “A” distance requires further refinement. These findings support efforts to standardize US examination protocols and promote consensus in diagnostic methodology, though further research is needed to address the remaining challenges.

## 1. Introduction

Rock climbing is gaining popularity and therefore injuries related to its practice have also spurred interest in both diagnosis and treatment fields [1]. Injuries to the annular finger pulleys and finger flexor tendons are common among rock climbers, representing a significant proportion of all injuries in this group [2]. The A2 pulley is the most frequently affected, followed by the A4, often resulting in damage to the fourth finger [3]. These injuries can be partial or complete and may involve one or more pulleys [4]. The multiple pulley ruptures are clinically evidenced by bowstringing of the flexor digitorum tendons [5].

The anatomy of this tissue, named annular (A) pulleys, is based on a digital fibro-osseous flexor sheath of the fingers [6], which are retinaculum portions that hold the tendons of the flexor digitorum superficialis (FDS) and flexor digitorum profundus (PDP) muscles against the phalanges [7]. This helps to optimize their biomechanical function and prevent bowstringing deformities, enabling the conversion of tendon excursion force into a suitable finger flexion motion pattern [5,8]. This system is known as the digital flexor pulley system and it comprises five annular pulleys (A1 to A5) and three cruciate pulleys (C1 to C3), with the A2 pulley being the most important [9]. The A2 pulley encircles the anterior and lateral aspects of the FDS and FDP tendons, inserting on both sides into the periosteum of the proximal phalanx. It spans the proximal and middle third of the proximal phalanx and is the longest of the pulleys, ranging from 16.8 mm [10] to 20 mm [11]. Its thickness ranges from 0.3 mm to 0.7 mm [12] but tends to be thicker in experienced climbers at 1.2 mm [13]. According to biomechanical studies, the A2 pulley is the strongest of the finger pulleys [14].

High-resolution ultrasound imaging (US) is widely regarded as the gold standard for detecting annular finger pulley injuries [15]. Pulleys can be diagnosed via US by direct signs and indirect signs [7]. The direct sign is to visualize the injury directly without the need to perform an indirect measurement that estimates the degree of rupture. For this, the visualization of the tissue in question should be optimal, and, fortunately, high-frequency US allows a complete visualization (100%) of A1, A2 and A4 pulleys [16]. Although direct signs are being used more and more due to the higher resolution of US equipment, the most recognized and extensively studied US variable remains the tendon–bone distance (TBD) indirect sign [17]. Clear discrepancies were found across the studies focusing on the level of the proximal phalanx over which TBD is measured to diagnose A2 pulley tears [18]. Such anatomical landmarks varied from proximal to distal among the proximal-medial third of the proximal phalanx [19], the central point of the proximal phalanx [20], the 15–20 mm distal from the proximal phalanx base [13], the distal third of the proximal phalanx [15] and the distal end of the A2 pulley [17]. These five anatomical landmarks can be simplified into two, as the first two are based on the midpoint of the proximal phalanx (MPP), and the remaining three are based on the distal edge of the A2 pulley (DA2).

So, in order to diagnose a rupture of the A2 pulley, the two anatomical landmarks for US TBD measurement that were most frequently mentioned in the current literature were the MPP and DA2 [18]. Thus, establishing the reliability and validity of US for identifying such anatomical landmarks to carry out the TBD measurement afterwards has important clinical utility to avoid controversies among sonographers and also to differentiate between partial and complete A2 tears.

The main aim of this study was to determine the reliability and validity of US to identify and measure the midpoint of the proximal phalanx and the distal edge of the A2 pulley.

## 2. Materials and Methods

This was a cross-sectional study conducted on human cadavers. In total, 36 fingers (9 index, 9 middle, 9 ring and 9 little fingers) from 9 hands of 5 fresh frozen human cadaver arms (average age of 77, range of 74 to 80 years) were studied. The specimens had no signs or history of finger, hand or wrist injuries or surgery and were left to thaw at room temperature before dissection. All specimens were obtained from bodies donated to the Faculty of Medicine and Health Sciences (Clinic Campus) at the University of Barcelona. Institutional review board approval was obtained prior to the study. The cadaver tissues used were part of a body donation program and were in compliance with current Spanish legislation about ethics in research. None of the specimens showed trauma, deformities or surgical scars on the hand.

For US examination, we used a Canon Aplio i800 US machine equipped with a 22 MHz ultra-high-frequency hockey stick (i22LH8) and 24 MHz ultra-high-frequency iDMS linear transducer (i24LX8) (Canon medical system^®^, Tustin, CA, USA). Abundant US gel was used to avoid compression of the finger by the transducer. The finger examination position was 0° or the neutral position of the metacarpophalangeal (MCP), proximal interphalangeal (PIP) and distal interphalangeal (DIP) joints with the hand in the supine position.

**Figure 1:**
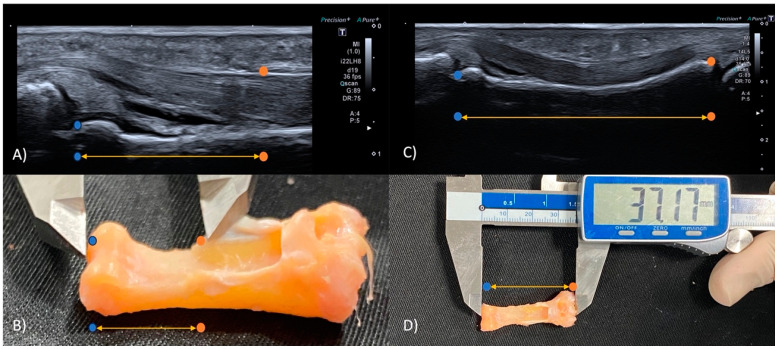
PP distance and “A” distance. (**A**) “A” distance by US in long axis, orange point is the distal edge of the A2 pulley and the blue point is the distal edge of the proximal phalanx; (**B**) “A” distance by caliper; (**C**) PP distance by US in long axis, orange point is the proximal edge and the blue point is the distal edge of the proximal phalanx; (**D**) PP distance by caliper. In (**B**,**D**) figures, the A2 pulley is cut to avoid altering the measurement values.

The two anatomical references for later measuring TBD are the MPP and the DA2. For the first landmark, the length of the proximal phalanx (PP distance) was measured with the linear transducer (Figure 1C,D) and, afterwards, estimated half of such length. For the second landmark, as it is not possible to directly identify the distal edge of the A2 pulley if there is a rupture of it, the following method was applied: to measure the distance between the distal edge of the proximal phalanx and the distal edge of the A2 pulley, called the “A” distance (Figure 1A,B), on the healthy contralateral side. The stick transducer was used to measure the “A” distance.

Both measurements were performed by two experienced sonographers, observers 1 (O1) and 2 (O2), who were blinded to each other and to the previous randomization process of the sample. Then, all fingers were dissected until only the proximal phalanx was left with its respective A2 pulley and the same measurements were performed with a digital caliper (Qfun^®^ digital caliper, Hong Kong, China, 0–150 mm, CN) by a blinded third observer (O3).

Each of the 36 fingers was tested by 3 raters and the order of testing was chosen by block randomization. Each block had a fixed size of three (A = O1, B = O2, C = O3), but the US examination was necessarily performed before dissection measurement, so two randomization order possibilities were available: ABC and BAC. The first of the two sequences was chosen at random. When three fingers had been included, the process was repeated until the desired sample was achieved. The three raters were blinded to the testing process.

Data are described using the most appropriate statistics for the nature and scale of measurement of each variable: mean, standard deviation and the range between the maximum and minimum value. For reliability among the three observers, the Intraclass Correlation Coefficient (ICC) and the Lin-s concordance correlation coefficient (CCC), along with the Bradley–Blackwood test. For validity, Passing–Bablok regression was used. T student and ANOVA repeated measures procedure was employed to compare the differences between both measurements. Data were analyzed using Stata SE for Windows (Stata Corp^®^. 2021. Stata Statistical Software: Release 16. College Station, StataCorp LLC, College Station, TX, USA). Significance was set at *p* < 0.05.

## 3. Results

A total of 36 fingers were measured by 3 observers blinded to the testing process and to each other. O1 and O2 performed the US measurements independently and O3 completed the same measurement by a caliper. These details are provided in Table 1.

The differences in the measurements of the PP distance and the “A” distance between both sonographers, O1 and O2, are very small, whereas the comparison of these with the caliper measurement, O3, is bigger. The US measurements tend to be shorter than the measurements by the caliper.

The reliability between the three observers was high for the PP distance measurement, at CCI > 0.75. In order from highest to lowest values in accordance with the individual and average CCI, the reliability scores were as follows: O1 vs. O2 (0.98, 0.99), O2 vs. O3 (0.94, 0.97) and O1 vs. O3 (0.93, 0.96). The highest level of agreement was obtained between both sonographers, O1 and O2, with a Rho value of 0.98 and a non-significant Bradley–Blackwood *p*-value of 0.153. Although the concordance between sonographers and caliper measurements was high according to CCI, the Bradley–Blackwood *p*-value was significant, which means lower reliability. For the PP distance measurement, systematic errors and proportional differences were found between the three observers by Passing–Bablok.

The reliability was lower for the “A” distance compared to the PP distance values. Even if the agreement between O1 and O2 was high (0.79, 0.89), it was low between US measurements and the caliper measurements, O1 vs. O3 (0.27, 0.43) and O2 vs. O3 (0.30, 0.46). For the “A” distance measurement, systematic errors and proportional differences were found between the three observers by Passing Bablok. The smallest proportional difference was found between O1 and O2 (B = 1.02). The interobserver reliability values are provided in Table 2.

**Figure 2:**
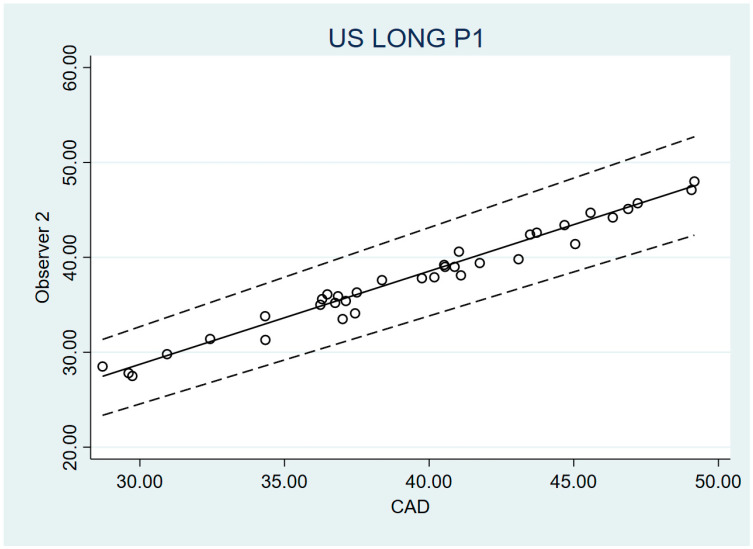
Bland–Altman graphic of PP distance O2 vs. O3.

**Figure 3:**
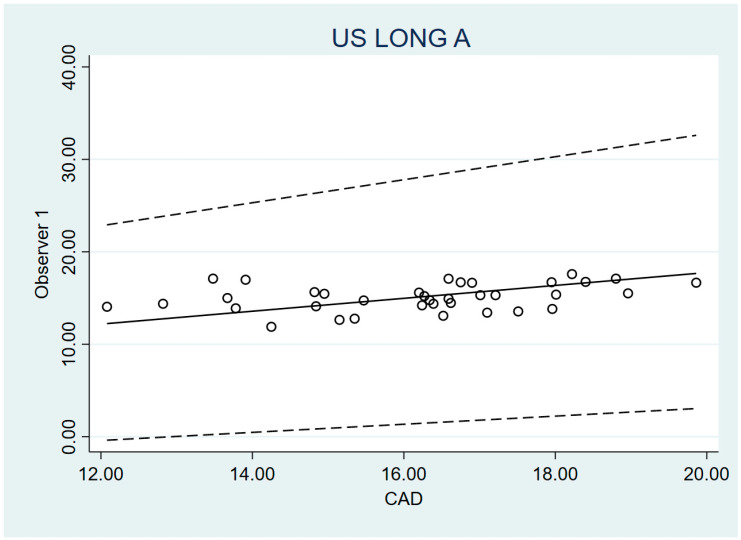
Bland–Altman of A distance O1 vs. O3.

The validity of the US measurements of PP and “A” distances were compared to the anatomic measurements by a caliper, which is considered the gold standard. For this purpose, the Passing–Bablok method was used. In the four analyses carried out, both the value 0 (for the 95% CI of A) and the value 1 (for the 95% CI of B) were within the intervals. So, no systematic and proportional errors were found. Thus, no significant differences were established between the US and caliper measurements, neither in PP distance nor in A distance. However, in contrast to the PP measurement, “A” distance measurement had very wide 95% CI intervals and Lin’s concordance was weak. Therefore, in the case of PP distance measurement, it can be concluded that the caliper measurement can be substituted for the US measurement. On the other hand, in the “A” distance measurement, such validity is significant but lower. The validity values are provided in Table 3 and Figure 2 and Figure 3.

Differences among PP and “A” distances between the three observers were analyzed by T-student for related samples and the repeated measures ANOVA. There is a significant difference between PP distance value differences between O1, O2 and O3 compared to the “A” distance value differences between O1, O2 and O3. The differences between O1 and O2 are bigger for the “A” distance measurement (0.39 ± 1) than for the PP distance measurement (−0.28 ± 0.95). The differences between O1 and O3 are bigger for the PP distance measurement (−1.95 ± 0.14) than for “A” distance measurement (−1.15 ± 0.31). Thus, the “A” distance value differences are higher than the PP distance value differences between sonographers and lower between sonographers and dissection measurements by a caliper.

A unique exception was found in the differences between O2 and O3. There is no significant difference (−0.15 ± 0.30, Pr > 0.05) between averages of the difference in the PP distance between O2 and O3 (−1.66 ± 0.15) compared to the average difference obtained for the distance “A” between O2 and O3 (−1.50 ± 0.33). Therefore, there is an equivalence in the comparison among PP and “A” distances between O2 and O3. These values are provided in Table 4.

## 4. Discussion

To obtain a reliable US diagnosis of an A2 pulley rupture, both through direct and indirect signs, two crucial factors should be investigated thoroughly, as the effectiveness of US heavily depends on the skill of the sonographer. Firstly, it is essential to know the reliability of these measurements among sonographers and, secondly, to establish the validity of whether these US measurements correspond to real anatomy. Therefore, in this study, we aimed to determine the reliability and validity of US measurements compared to anatomical measurements on cadavers. Enhancing both anatomical visualization and reproducibility among sonographers would be highly beneficial. This improvement would lead to more accurate results and reduce discrepancies or disagreements among sonographers.

Descriptive values already show that measurements on anatomy using calipers are longer than those using US. This could confirm our suspicion that we cannot visualize the phalanx in its entirety with US [21]. This is not a problem when identifying the anatomical landmarks for TBD measurements, as even if the PP distance or “A” distance is shorter by US than in real anatomy, the identification of the anatomical landmark remains correct.

The reliability among sonographers is very high, even if the measurements are not identical. There are systematic errors between both sonographers, meaning that while the values are not identical, the measurements of the PP distance are very similar due to the consistent nature of the error. This might be because one of the sonographers was measuring the edges of the phalanx shorter and the other longer, but the error between them was maintained in all the measurements. On the other hand, the agreement between sonographers is lower for the “A” distance. This indicates that the variability among sonographers is lower for the PP distance than for the “A” distance. Therefore, measuring the TBD at MPP is more accurate than at DA2 based on the reliability values between sonographers to identify the anatomical landmarks. However, we suspect that for A2 pulley partial ruptures, the DA2 might be more appropriate. A 5 mm long pulley section from distal to proximal of the pulley, which simulates a small size partial rupture (20–25%) of the A2 pulley, did not provide a significant TBD increase at the MPP, unlike at the level of the DA2, in which the flexor tendon significantly raised from the distal third of the proximal phalanx [4]. So, DA2 could be a more appropriate anatomical landmark for diagnosing small partial ruptures of the A2 pulley by TBD measurement but less reliable than the MPP. Reliability between sonographers and caliper measurements is lower than among sonographers. Nonetheless, the reliability for measuring PP length is very high, indicating that despite differences, US can be reliable enough to identify the MPP anatomical landmark. For the measurement of “A” distance, the agreement values between sonographers and calipers are generally low.

The validity of the US measurements was obtained by comparing such values with caliper measurements. For PP distance measurements, it can be concluded that caliper measurements can be substituted with US measurements. This confirms that the MPP US measurement is highly valid even though the entire phalanx cannot be visualized with ultrasound [21]. The validity of the “A” distance by US is significant but is lower compared to PP distance measurements, so the accuracy of DA2 identification by US was not as high as expected. The significant but low reliability and validity values of the “A” distance measurement by US might happen for several reasons. Although US allows for an excellent depiction of the A2 pulley due to its fibrocartilaginous component that makes it thicker in the distal edge, the C1 pulley was only supposed to be seen in 45% of cases [16]. US image resolution has become higher in recent years, which enables us to obtain higher image quality but also tissues that were not able to visualize before as the C1 pulley. So, a better C1 pulley visualization can raise questions as to where the boundary between pulley A2 and C1 is. Anatomic variation of the finger flexor pulley system is widely known and it also increases the difficulty of pulley visualization by US [10,22].

There is currently no agreement on the optimal measurement protocols or specific TBD reference values to diagnose a pulley injury, especially due to the heterogeneity of the anatomical landmark used [18]. Now that this controversy has been studied and reflected by the present study, it is time to start standardizing and reaching a consensus on other controversial factors of the ultrasound examination as coupling agent, finger position and degree of flexor activation force used [18]. Further research is needed in this direction to obtain a reliable and valid pulley rupture diagnosis.

The primary limitation of this study is the small sample size. It is important to interpret the findings carefully since all measurements were conducted on in vitro specimens. The main concern is that the reliability and especially the validity of measuring the “A” distance by US are not high, despite its apparent clinical usefulness as an anatomical landmark. Additionally, another potential limitation is that reliability and validity values were assessed across all triphalangeal fingers, without comparing the index, middle, ring and little fingers between them.

US, considered the gold standard tool in diagnosing pulley ruptures, is reliable and valid for identifying anatomical landmarks such as the midpoint of the proximal phalanx and the distal edge of the A2 pulley. This is crucial for accurately measuring the tendon–bone distance and thereby achieving a reliable diagnosis. Moreover, this can facilitate consensus on measurement methodology, thereby reducing current issues related to methodological heterogeneity and variability in TBD results. However, while the reliability and validity of the “A” distance measurement are significant, they are lower compared to the PP distance. Therefore, further research and understanding are necessary in this regard.

## Figures and Tables

**Table 1:** Baseline measurements.

Measurement	Observers	Mean ± SD	Range
PP distance	O1 US	37.5 ± 5.3	27.7–47.8
	O2 US	37.8 ± 5.4	27.5–48
	O3 CAL	39.5 ± 5.5	28.7–49.2
“A” distance	O1 US	15.1 ± 1.5	11.9–17.6
	O2 US	14.7 ± 1.8	10.8–18.3
	O3 CAL	16.2 ± 1.8	12.1–19.9

SD, standard deviation; US, ultrasound; CAL, caliper; PP, proximal phalanx; “A”, between distal edges of PP and A2 pulley.

**Table 2:** Interobserver reliability values.

	PP Distance		“A” Distance	
	ICC Individual (95% CI) Average (95% CI)	Lin Coef. Rho (95% CI) *p*-Value	ICC Individual (95% CI) Average (95% CI)	Lin Coef. Rho (95% CI) *p*-Value
O1 vs. O2	0.98 (0.97, 0.99)	0.98 (0.97, 0.99)	0.79 (0.62, 0.89)	0.79 (0.68, 0.91)
	0.99 (0.98, 1.00)	0.153	0.89 (0.76, 0.94)	0.007
O1 vs. O3	0.93 (0.01, 0.98)	0.92 (0.89, 0.96)	0.27 (−0.02, 0.54)	0.27 (0.02, 0.52)
	0.96 (0.03, 0.99)	0.000	0.43 (−0.05–0.70)	0.002
O2 vs. O3	0.94 (0.11, 0.99)	0.94 (0.91, 0.97)	0.30 (−0.03, 0.57)	0.29 (0.06–0.52)
	0.97 (0.20, 0.99)	0.000	0.46 (−0.06, 0.73)	0.000

ICC, interclass correlation coefficient; CI, confidence interval; Lin Coef, Lin-s concordance correlation coefficient.

**Table 3:** Validity values between ultrasound and caliper measurements.

Comparison	Variable	Regression Line	Cusum Test
		A (95% CI) B (95% CI)	H *p* value
“A” Distance O1 vs. O3	Y: O1	3.81 (−5.68–7.88) 0.70 (0.44–1.24)	0.92 >0.20
	X: O3		
“A” Distance O2 vs. O3	Y: O2	−2.37 (−13.53–4.83) 1.02 (0.62–1.75)	0.92 >0.20
	X: O3		
PP Distance O1 vs. O3	Y: O1	−1.23 (−3.48–1.58) 0.98 (0.91–1.04)	1.15 0.10–0.15
	X: O3		
PP Distance O2 vs. O3	Y: O2	−0.70 (−3.28–1.38) 0.98 (0.93 ± 1.04)	0.69 >0.20
	X: O3		

**Table 4:** Differences among PP and “A” distance between the three observers.

	Mean ± SD	95% CI	Pr Value
O1 vs. O2			
PP dist diff	−0.28 ± 0.95	(−0.61, 0.03)	
A dist diff	0.39 ± 1	(0.05, 0.73)	
PP dist vs. A dist	−0.68 ± 1.22	(−1.09, −0.26)	0.00
O1 vs. O3			
PP dist diff	−1.95 ± 0.89	(−2.25, −1.65)	
A dist diff	−1.11 ± 1.90	(−1.76, 0.46)	
PP dist vs. A dist	−0.84 ± 1.73	(−1.42, −0.25)	0.00
O2 vs. O3			
PP dist diff	−1.66 ± 0.92	(−1.97, −1.35)	
A dist diff	−1.50 ± 1.99	(−2.18, −0.83)	
PP dist vs. A dist	−0.15 ± 1.80	(−0.77, 0.45)	0.60

## Data Availability

The original contributions presented in the study are included in the article, further inquiries can be directed to the corresponding author.
